# 1004. Cladophora in Lake Michigan May Serve as Important Reservoirs for Antibiotic-Resistant Bacteria

**DOI:** 10.1093/ofid/ofab466.1198

**Published:** 2021-12-04

**Authors:** Dannielle C Grayer, Latania K Logan

**Affiliations:** 1 Rush University Medical Center/Rush University Children’s Hospital, Chicago, Illinois; 2 Rush University Medical Center, Chicago, IL

## Abstract

**Background:**

Cladophora is a green algae, native to the Great Lakes, and found in large quantities along Lake Michigan shorelines. Previous studies have shown that Cladophora provide protection and nutrients for the Enterobacteriaceae (Ent) family, allowing persistence and regrowth. Chicago waterways harbor concerning antibiotic-resistant (AR) Ent, however the community reservoirs are unknown. Our primary objective was to assess whether Cladophora harbor AR Ent and to secondarily assess AR Ent in local beach waters where Cladophora are present.

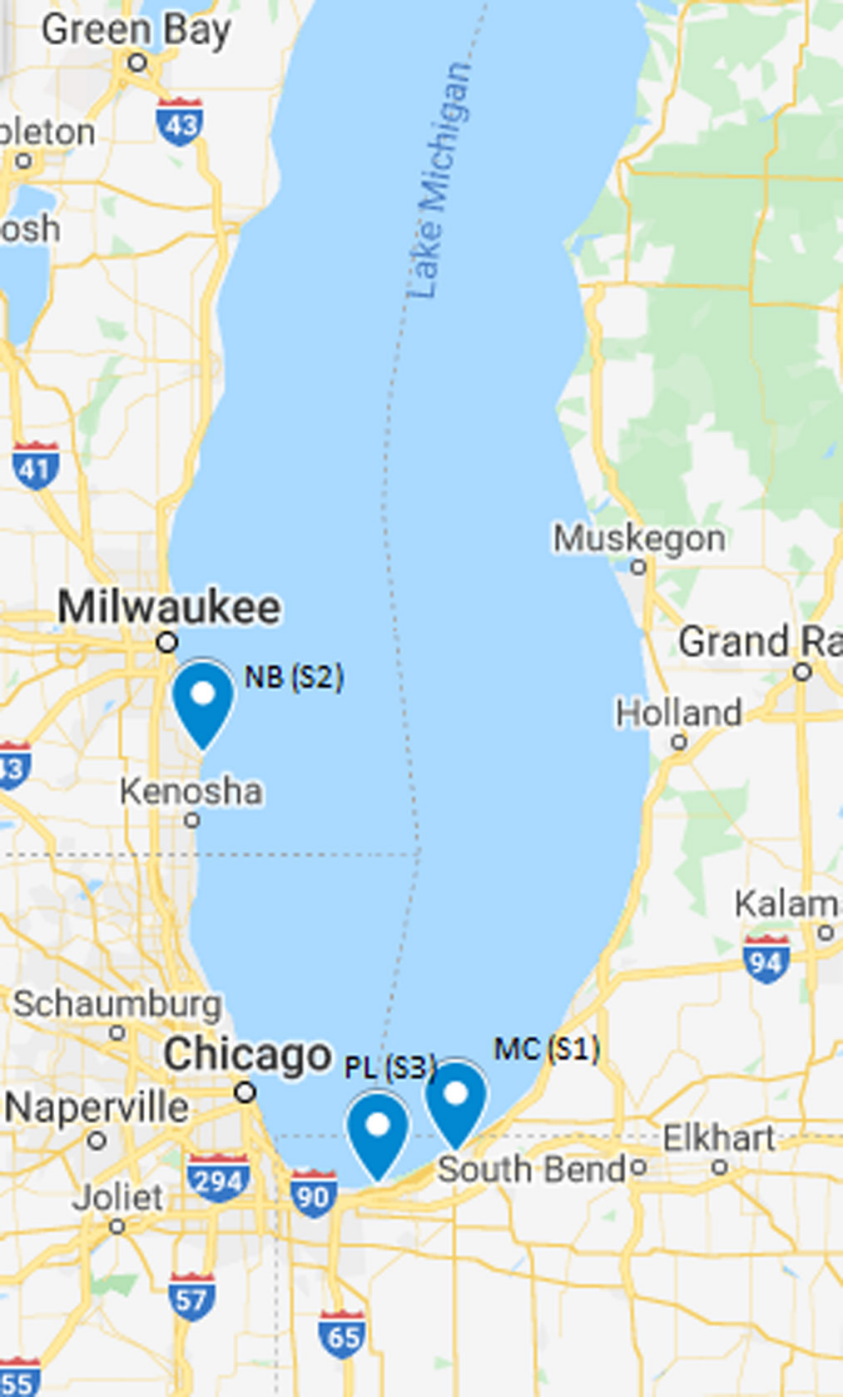

Figure 1. Map of Lake Michigan showing sites (S1-S3) where Cladophora samples were collected. NB, North Beach, Racine, Wisconsin; Michigan City, Indiana; PL, Portage Lakefront, Indiana Dunes National Park, Indiana.

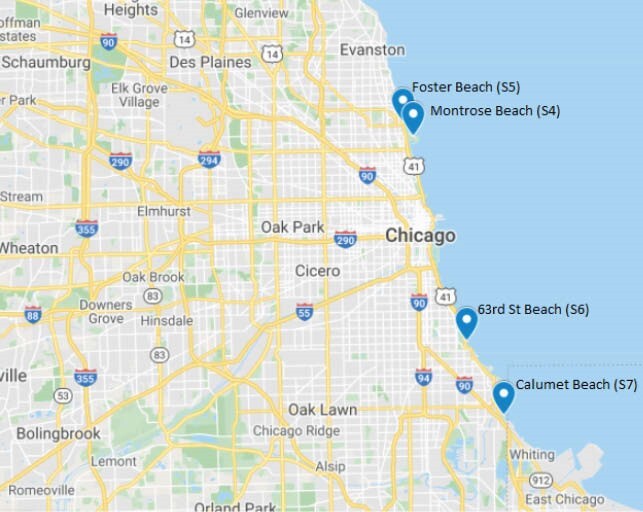

Figure 2. Map of Lake Michigan showing sites (S4-S7) where beach surface water samples were obtained. Montrose, Foster, 63rd St, & Calumet beaches in Chicago, Illinois.

**Methods:**

Cladophora were processed from three Indiana Lake Michigan sites (S1 and S2; Fig 1) in 2002 and 2012 (S3; Fig 1) at the USGS Lake Michigan Ecological Research Station (Chesterton, IN). In 2015, surface water samples were obtained by the USGS at four Chicago beaches (S4-S7; Fig 2), which also amass Cladophora. Bacteria were isolated shortly after collection. In 2019-2020, Ent were cultivated and susceptibilities were performed at Rush.

**Results:**

In 2002-2003 (S1 and S2), 160 *E. coli* were cultured from Cladophora. There was AR to multiple classes, highest overall in tetracyclines (7.5%, range 6.2%-18.7%), cefoxitin (8%), and cefazolin (5.6%). Resistance to cefuroxime was 0.6%. Four Salmonella isolates from 2012 (S3) were pan-susceptible, while two Citrobacter isolates were resistant to penicillins, 1^st^ and 2^nd^ generation cephalosporins, and cephamycins. Beach surface water samples from 2015 revealed more pronounced AR in *E. coli* (n=185) involving multiple classes, including highest in ampicillin (12.4%), tetracyclines (8.1%); piperacillin (7%); cefazolin (3.8%), cephamycins (3.2%) and amoxicillin-clavulanate (2.7%). Resistance to 3^rd^-generation cephalosporins, fluoroquinolones, trimethoprim/sulfamethoxazole ranged from 0.5-2%. AR Ent varied by beach site with highest percentages at S4, the only site with an associated dog beach.

**Conclusion:**

These findings suggest that Cladophora in recreational waterways may serve as reservoirs for AR Ent. Differences in AR Ent at beach sites may reflect varying degrees of fecal contamination. Identifying community reservoirs is key to better understanding the acquisition of antibiotic resistant Ent among healthy populations and has long-term ecological and public health implications.

**Disclosures:**

**All Authors**: No reported disclosures

